# Characterizing the Long-term Care and Community-dwelling Elderly Patients' Use of the Emergency Department

**DOI:** 10.7759/cureus.3642

**Published:** 2018-11-26

**Authors:** Sachin Trivedi, Christopher Roberts, Erwin Karreman, Kish Lyster

**Affiliations:** 1 Emergency Medicine, University of Saskatchewan, Saskatoon, CAN; 2 Miscellaneous, Regina Qu'appelle Health Region, Regina, CAN; 3 Internal Medicine, Regina Qu'appelle Health Region, Regina, CAN

**Keywords:** geriatrics, patient flow, emergency medicine, emergency medical services

## Abstract

Introduction

Elderly patients, particularly those in long-term care (LTC), are a growing proportion of patients who present to the emergency department (ED). This population is medically complex, with high burdens on ED resources and patient flow. This study sought to characterize how elderly LTC and community-dwelling (CD) patients use ED services.

Materials and methods

This was a retrospective cohort study that assessed approximately 200 senior (age>65) ED visits. These patients were either residing in LTC facilities or they were CD. All participants lived in the same, medium-sized Canadian city. Data indicating demographic information, acuity of presentation, and administrative parameters (such as disposition status or length of stay) were collected and analyzed.

Results

A few statistically significant differences between the populations were noted. This included mean age, which was 82.6 years in the LTC population and 77.3 for the CD group (p<0.001). There were 27 repeat visits among patients in the LTC group, compared to six from the CD patients (p<0.001). In the LTC population, 75 patients required transport from emergency medical services (EMS) compared to 41 from the control group (p<0.001).

Conclusion

LTC patients re-present to the ED and use EMS services more frequently than their CD counterparts. This difference indicates potential areas to target for future quality improvement work to help enhance care to this vulnerable population.

## Introduction

The care of the geriatric population can be fraught with challenges due to medical and socioeconomic factors. The emergency department (ED) is often the point of first contact for many elderly persons, and it is a difficult place for these patients to be cared for appropriately. Multiple studies have shown that the elderly population is increasingly using the ED [1-5]. There is a disproportionate growth in the number of older patients presenting to the ED and requiring hospital admission [2-3]. Adding to the complexity of this patient population is the knowledge that older adults can have ambiguous concerns or atypical presentations of common complaints [6]. The use of diagnostic testing, consultation, length of stay (LOS), and hospital admission has been shown to increase with age in Canadian EDs [7-8]. Patients over the age of 85, who are at increased risk for adverse events while in the ED, are more likely to experience prolonged ED LOS [9]. There is also data to suggest that this population is at high risk to re-present to the ED, and that a previous admission is a strong predictive factor for readmission [3-4]. An American study has shown that the LOS of ED visits will increase and that interventions such as expanding ED size may be required to keep up with the increased demand [5].

Broadly speaking, the aging population can be considered as either those who are community-dwelling (CD) and those who reside in long-term care (LTC). When looking at this demographic, a central question is how to provide patient-centered emergency care to the LTC population. These patients are unable to independently maintain their activities of daily living due to different disease states. With this in mind, the LTC population can be thought of having higher baseline care needs than their CD counterparts. Historically, there have been debates about the appropriateness of LTC patients who are referred from their facilities to present to the ED [10]. It has been shown that patients who meet specific criteria may have had their visit to the ED prevented [11]. A Canadian study looked specifically at all of the LTC facilities in Ontario and their use of the ED, demonstrating these residents place a heavy demand on health care resources, citing admission rates and emergency medical services (EMS) usage as being particularly high [7]. In Australia, residents of aged care facilities faced a considerable burden when transferred to the ED [8]. This included high rates of investigation and intervention and increasing rates of complications like delirium and hospital-acquired infections. Poor bilateral communication patterns between the LTC facilities and the ED is a known problem, which frequently leaves LTC patients dissatisfied at the end of their experience [12-15].

There is a clearly demonstrated need for improved geriatric care, notably for those presenting to the ED [16]. Some initial data from interventions with geriatrics-focused practitioners have demonstrated the potential to reduce transfers to the ED and hospital admissions [17-21]. However, there remains a paucity of data comparing the specifics of the use of emergency services by the LTC and CD elderly populations. The goal of this study is to compare the characteristics of how these two populations are utilizing the ED. Understanding the differences in ED utilization may help identify potential areas to intervene on in order to enhance the quality of care delivered to this population.

## Materials and methods

Study design and setting

This study was a retrospective cohort study comparing two groups of patients aged 65 and older; those who resided in LTC facilities and those CD. The LTC facilities that data was collected from were those operated by the local health region. There are a total of nine health region-run LTC facilities. Across all these facilities, there are 1, 337 beds. These were selected based on their unique postal codes. Data was collected from the only two hospitals with EDs in Regina, Saskatchewan.

Sample selection and data abstraction

A convenience sample of approximately 100 charts for each of the sample groups was selected. This sample size was selected, as it was felt to provide a general overview of local ED utilization trends. Charts were collected if they were deemed to meet our inclusion criteria. For the CD group, this was if a patient was older than age 65. In addition to this age cut-off, the inclusion criteria for the LTC group required that the patient reside in a facility, as delineated by the unique residential postal codes. The first 100 presentations from each of two EDs in Regina beginning in January 2012 were collected. An attempt was made at abstracting data from a consecutive sample, however, if a given chart was unavailable, the next available presentation meeting criteria were provided by the Health Records department. Repeat presentations from the same patient were documented as unique visits and distinguished for further analysis. Any personal care home or private facility that was not run by the local health region was deemed to be part of the CD population. This was due to a lack of standardization of care with the care protocols from the health region operated facilities.

Data regarding age (years), sex (male or female), Canadian Triage and Acuity Scale (CTAS) score (1 - 5), method of arrival (EMS or private vehicle), and disposition were collected. Disposition was categorized as admitted, discharged, or left without being seen (LWBS). LOS data was acquired from the Regina Qu'Appelle Health Region (RQHR) Health Records department.

Data abstraction was performed by a single reviewer (ST). A standardized, password-encrypted, electronic spreadsheet was designed in which to input the data. The metrics chosen to collect the data were all deemed to be objective and not up to the interpretation of the reviewer. Data were statistically analyzed using IBM SPSS Statistics 22 and included descriptive statistics, Chi-square, and independent t-tests where appropriate. A p value of <0.05 was deemed to show statistical significance.

Ethical approval was received by the RQHR Research Ethics Board (REB 13-110).

## Results

In total, 100 visits from the LTC and 99 visits from the CD populations were analyzed. One visit from the CD population was excluded due to relevant data being undocumented within the chart. Table 1 describes demographic information of our samples.

**Table 1:** Comparative demographics of long-term care and community-dwelling cohorts LTC: long-term care; CD: community dwelling

Characteristic	LTC Sample (n = 100)	CD Sample (n = 99)	Significance level (p)
Sex			
Male	46	44	
Female	54	55	0.83
Age			
65-70	14	29	
71-80	25	33	
81-90	40	31	
>90	21	6	
Mean age (yrs)	82.6	77.3	<0.01
Visit Statistics			
Single visit	73	93	
Repeat visits	27	6	<0.01

Table 2 illustrates comparisons between the two groups for ED-related variables. In the LTC population, 75 visits were documented as requiring transport from EMS compared to 41 from the control group (p<0.01). Dispositions were not statistically different between the two populations with 50 patients from LTC and 43 from the CD group being admitted. Additionally, CTAS distributions across both groups were statistically similar (p=0.13).

**Table 2:** Comparison of selected characteristics EMS: emergency medical services; LWBS: left without being seen; CTAS: Canadian Triage and Acuity Scale

Characteristic	LTC Sample (n = 100)	CD Sample (n = 99)	Significance Level (p)
Arrival to ED			
Brought in by EMS	75	41	<0.01
Private Vehicle	25	57	
Unknown	0	1	
Disposition			
Admitted	50	43	0.23
Discharged	46	55	
LWBS	4	1	
CTAS Score			
1	5	1	
2	9	21	
3	43	44	
4	33	22	
5	10	10	

Tables 3-4 show admission CTAS or mode of arrival stratified by CD/LTC. With respect to the CTAS scores, a significantly higher proportion of CTAS 1-3 patients were admitted in both samples. In terms of mode of arrival, there was a significant difference in those from the CD population arriving by EMS getting admitted compared to those who arrived by private vehicle. This difference was not noted among the LTC population. Table 5 shows CTAS by mode of arrival. Again, a significant difference was noted in the CD population but not the LTC group.

**Table 3:** Disposition by CTAS classification for the CD and LTC cohorts CTAS: Canadian Triage and Acuity Scale; CD: community dwelling; LTC: long-term care

	Admitted	Not Admitted	Significance Level (p)
CD Sample			
CTAS 1-3	36	30	<0.01
CTAS 4-5	7	25	
LTC Sample			
CTAS 1-3	34	21	0.03
CTAS 4-5	16	25	

**Table 4:** Disposition by mode of arrival for the CD and LTC cohorts CD: community dwelling; LTC: long-term care; EMS: emergency medical services

	Admitted	Not Admitted	Significance level (p)
CD Sample			
EMS	30	11	<0.01
Private Vehicle	13	44	
LTC Sample			
EMS	41	33	0.33
Private Vehicle	9	13	

**Table 5:** CTAS classification by mode of arrival for both sample cohorts CTAS: Canadian Triage and Acuity Scale; CD: community dwelling; LTC: long-term care; EMS: emergency medical services

	EMS	Private Vehicle	Significance Level (p)
CD Sample			
CTAS 1-3	33	33	0.02
CTAS 4-5	8	24	
LTC Sample			
CTAS 1-3	45	12	0.29
CTAS 4-5	30	13	

Finally, LOS metrics were abstracted. In the LTC population, patients categorized as CTAS one to three had a median LOS of five hours. In the respective CD group, LOS was five hours. Those triaged as CTAS four or five were found to have a median LOS of three hours. The comparative CD group was recorded as having a median LOS of four hours. For both patient groups, any patient who was admitted had a median LOS of six hours.

## Discussion

Our study sought to compare ED presentations among those elderly in the community and those from LTC so that areas with discrepancies may be targeted for interventions to improve patient care and flow. Significant differences were noted in patient age, repeat presentation to the ED, and the use of EMS.

Additionally, CTAS scores, length of stay, and admission rates were examined. The LTC group was noted to have lower acuity scores although this comparison did not reach statistical significance. Lengths of stay and admission rates by CTAS were also statistically similar. The data suggest that the patients are managed appropriately and the disposition determined in a similar fashion regardless of where patients reside.

LTC patients were found to be significantly older than community dwellers. While this is a non-modifiable component, it is of importance regarding clinical care and the use of resources. Studies have shown that increasing age results in a heavier burden of resources [7-9]. As well, it has been shown that the older patients have a higher likelihood of being admitted to the hospital, with increased length of stay [2-3,7-9]. While there was no statistical difference in admission rates, there did appear to be a trend towards more admissions in the LTC cohort.

There were a higher number of patients found to return to the ED amongst the LTC group. While details of subsequent re-presentations were not investigated, it identified an area where improvements may reduce the ED burden. Communication between LTC homes and emergency physicians have been previously shown as suboptimal [12-15]. Previous literature has demonstrated that significant information gaps are present when LTC patients are transferred to the ED [22]. There has also been evidence demonstrating a clear discrepancy between the information desired by ED staff and that which is provided from LTC [23]. As such, improved handover protocols may represent an area for intervention. Furthermore, in cases with additional resources, such as multidisciplinary management teams, patient outcomes have been improved [18,21]. As a result, clear and direct physician communication, with additional care team members, has the potential to decrease these repeat presentations that bog down patient flow and decrease patient satisfaction.

Perhaps the most reasonable area for intervention surrounds the use of EMS by LTC patients. It has been indicated that older age is an independent risk factor for transportation to the ED via EMS [24]. Our data identified that LTC patients present to the ED via EMS at a significantly higher volume, consistent with previous literature [7]. However, we reported a statistically significant difference in EMS use by LTC patients and CD patients. CD elderly used EMS at a higher frequency when associated with a higher CTAS. Significantly higher rates of admission after EMS use were also documented in the CD sample. This is a reasonable expectation but was not reflected in the LTC group. This data suggests that LTC patients might be using EMS when not appropriate. This could be due to the LTC staff being unfamiliar with options or limited transportation modalities available to patients and their families. Pilot studies show mobile registered nurses or EMS treating patients onsite have reduced presentations to the ED [20-21,25]. Together, increased access to multidisciplinary teams and the increased management of patients in LTC facilities by medical providers may have the potential to reduce ED visits and improve patient flow.

Our study was limited by different factors. First, as a convenience sample that looked at a limited number of presentations, there is an inherent risk of selection bias. Furthermore, as a retrospective chart review, there is always a risk of erroneously recorded information. In order to mitigate this risk, only information that was felt to be standardized was collected. As a direct result of this standardization attempt, we were unable to collect information regarding the comorbid status or frailty of the enrolled patients and the exact reason for the repeated presentations to the ED. As well, we did not have a second reviewer assess the patient charts to ensure inter-rater reliability. With respect to the length of stay, we found discrepancies between what was provided to us by the Health Records Department and the actual paper charts, as information was often missing from the paper charts. As such, it was assumed that the information provided by Health Records was reliable data because it was consistently reported.

## Conclusions

Elderly populations, including those from LTC, are becoming an increasing burden on ED resources and patient flow. Notable differences were recognized in LTC patients – advanced age, higher re-presentation rates to the ED, and increased use of EMS services. With this in mind, future research should be directed at the reasons for ED revisits. Interventions aimed at improving handovers and reducing EMS use should also be considered.
